# 3D organization of telomeres in porcine neutrophils and analysis of LPS-activation effect

**DOI:** 10.1186/1471-2121-14-30

**Published:** 2013-06-26

**Authors:** Florence Mompart, David Robelin, Chantal Delcros, Martine Yerle-Bouissou

**Affiliations:** 1 INRA, UMR 444, Génétique Cellulaire, F-31326 Castanet, Tolosan, France; 2 Université de Toulouse, INP, ENVT, UMR 444, Génétique Cellulaire, F-31076, Toulouse, France

**Keywords:** Neutrophils, Telomere Associations, LPS-activation, 3D-FISH, Nuclear Architecture

## Abstract

**Background:**

While the essential role of 3D nuclear architecture on nuclear functions has been demonstrated for various cell types, information available for neutrophils, essential components of the immune system, remains limited. In this study, we analysed the spatial arrangements of telomeres which play a central role in cell fate. Our studies were carried out in swine, which is an excellent model organism for both biomedical research and agronomic applications. We isolated bacterial artificial chromosome (BAC)-containing subtelomeric p and q sequences specific to each porcine chromosome. This allowed us to study the behaviour of p and q telomeres of homologous chromosomes for seven pairs chosen for their difference in length and morphology. This was performed using 3D-FISH on structurally preserved neutrophils, and confocal microscopy. Resting and lipopolysaccharide (LPS)-activated states were investigated to ascertain whether a response to a pathogen aggression modifies this organization.

**Results:**

The positions of the p and q telomeres relative to the nuclear outer border were determined in the two states. All p telomeres changed their position significantly during the activation process, although the effect was less pronounced for the q telomeres. The patterns of telomeric associations between homologs and their frequencies were analysed for 7 pairs of chromosomes. This analysis revealed that the distribution of pp, qq and pq associations differs significantly among the 7 chromosomes. This distribution does not fit with the theoretical distribution for each chromosome, suggesting that preferential associations occur between subtelomeres.

**Conclusions:**

The percentage of nuclei harbouring at least one telomeric association between homologs varies significantly among the chromosomes, the smallest metacentric chromosome SSC12, which is also the richest in gene-density, harbouring the highest value. The distribution of types of telomeric associations is highly dependent on the chromosomes and is not affected by the activation process. The frequencies of telomeric associations are also highly dependent on the type of association and the type of chromosome. Overall, the LPS-activation process induces only minor changes in these patterns of associations. When telomeric associations occur, the associations of p and q arms from the same chromosome are the most frequent, suggesting that “chromosome bending” occurs in neutrophils as previously observed in gametes.

## Background

Considerable progress in the knowledge of the genome of several species has been achieved with the release of complete sequences giving new information about mechanisms controlling gene expression. However, genetic modifications are not sufficient to explain all phenotypes observed and gene expression can be modified by epigenetic factors. These epigenetic factors can influence the organization of chromatin conformation and chromatin loops in the nucleus as well as their interactions with other nuclear compartments. Numerous data have convincingly proven that there is a link between 3D genome organization in the nucleus and its function [1-3] and that the position of chromosomes in the nucleus plays an important role in gene regulation. In this context, studies of the nuclear topography of whole chromosome territories in various cell types have revealed their non-random organization (for reviews, see [2,4,5]). Telomeres, the end of chromosomes, have been particularly studied because of their essential role (reviewed in [6-8]). They consist of hexameric nucleotide sequences that are repeated hundreds or even thousands of times (TTAGGG in humans) coated by a group of proteins, termed shelterin. Intact telomeres prevent end-to-end fusions and degradation of the chromosome ends and contribute to appropriate chromosome positioning within the nucleus [6]. They demonstrate an intrinsic tendency for clustering as observed in mammalian nuclei both in somatic cells [9-13] and gametes [14,15]. In plants and animals, during early meiosis all or most telomeres cluster to a single region on the nuclear membrane [16], probably because of their key roles in meiosis in chromosome pairing and recombinogenic processes (reviewed in [17]). Telomeres were also found clustered at the nuclear periphery in human sperm [14,15], in yeast where they are known to form large silencing clusters [18-20], and in Drosophila [21]. They were shown to be organized in a non-random, cell-type and cell-cycle dependent manner in somatic cells [10,11,13,22]. With the help of controlled light exposure microscopy, it has been shown that telomeres share small territories positioned at the interface of chromatin domains on living cells, where they associate dynamically [23].

It is now clearly demonstrated that telomeres assume a regulated order in the nuclei of normal cells and that their aberrant organization is linked to the initiation and dynamic propagation of genome instability, a hallmark of various diseases including cancer [24,25]. It has been observed that normal and tumour cell nuclei differ in their 3D telomeric organization, with differences including modifications in the spatial organization, in numbers and size [26]. These changes have been used to define the transition of mononucleated H-cells to multi-nucleated Reed-Sternberg cells, the latter being the diagnostic cells in Hodgkin lymphoma [27,28].

Telomere is maintained primarily by telomerase. In the absence of this enzyme, telomeres shorten with cell divisions. In human nucleated blood cells, the average telomere length shows a highly significant decline with age that is most pronounced for the cells of the immune system. While telomere length in lymphocytes and granulocytes is quite similar early in life, it becomes increasingly shorter with age in lymphocytes compared to granulocytes [6]. Neutrophils, the major part of granulocytes, are terminally differentiated cells with a finite lifespan that do not express telomerase [29]. However in an inflammatory context, telomerase reactivation has been observed in these cells, prolonging their survival by delaying apoptosis [30].

We previously investigated the innate immune response in pig. Detailed knowledge of molecular and cellular events that occur in immune cells in response to pathogen infection is very important both for biomedical research, as swine serves as an important biomedical model for various human diseases, and for agricultural production [31,32]. We started with an analysis of the nuclear architecture of neutrophils which are, with monocyte-derived macrophages, the primary effector cells in severe infection. In response to a pathogen aggression, these immune cells undergo a series of distinct functional changes in state, including cytoskeletal rearrangements, reactivation of telomerase and variations in gene expression. Gathering more precise information on how telomeres are organized and interact in neutrophils and determining whether this organization changes during the activation process therefore remains of interest due to their essential role in nuclear architecture.

In an earlier study, we gave an overall view of the organization of chromosome territories, and we showed that telomeres also form clusters in spite of the polylobed structure of their nuclei [33]. However, the use of the (TTAGGG)_n_ repeat probe, which labels all the telomeres uniformly, did not allow us to define the nature of these telomeric associations nor to determine whether p and q telomeres of homologous chromosomes associate and, if they associate, how and at what frequency.

To answer these questions, we isolated BAC-containing subtelomeric sequences (Bacterial Artificial Chromosome) specific to p and q telomeres for each porcine chromosome to examine the spatial arrangements of telomeres and particularly the telomere associations in resting and in lipopolysaccharide (LPS)-stimulated neutrophils. For this purpose, we used exclusively three-dimensional fluorescence in situ hybridization (3D-FISH) on structurally preserved neutrophils in combination with confocal microscopy and 3D image analyses.

## Results

### Selection of BAC-containing subtelomeric sequences

While a complete set of human subtelomeric probes has been generated for use in FISH assays [34], such a collection was not available for swine. To construct one, we took advantage of the physical map of the swine genome generated by an international project using both high-throughput fingerprinting and BAC end sequencing. The project constructed a highly continuous BAC contig map covering 98% of the euchromatin of the 18 pig autosomes and the X and Y chromosomes [35]. On this map, accessible through pre-Ensembl, we selected the BAC clones situated closest to the telomeric extremity on both the p and q arms of each porcine chromosome. At least two BAC clones for each telomere were selected and their chromosomal locations were controlled by 2D-FISH on metaphases. We finally chose one BAC clone for each p and q telomere harbouring a specific, terminal and strong FISH signal (Additional file 1: Table S1; Additional file 2: Figure S1).

### Analysis of p and q telomere organization

The first step of the study consisted in determining whether, in immune cells, p and q telomeres of a specific chromosome join, how they join and at what frequency. To do so, we selected the p and q telomeric BAC probes of seven porcine chromosomes, chosen for their difference in length and morphology: **(1)** two submetacentric: SSC1 (*Sus scrofa domestica* 1 and SSC2), (**2)** one subtelocentric: SSC6, **(3)** two metacentric: SSC8 and SSC12, and finally **(4)** two telocentric: SSC13 and SSC17 chromosomes [36]. The p and q telomeric probes, specific to each chromosome pair, were cohybridized in 3D-FISH experiments on structurally preserved neutrophils. Two states (resting and LPS-activated) were analyzed to ascertain whether a response to a pathogen aggression modifies this organization. The neutrophils were activated using LPS, one of the best studied models for investigating innate immune response to infection with Gram-negative bacteria [37,38]. Under the conditions of our experiment, the cells are activated [33,39] but are not at the stage corresponding to the release of NETs (Neutrophil Extracellular Traps) [40,41], which would have been incompatible with our analysis. Indeed at this ultimate state, corresponding to cell death, the nuclear membrane is broken down and decondensed chromatin is released in the cytoplasm.

3D-FISH performed with the various subtelomere probes gave discrete signals in all experiments attempted. After each 3D-FISH experiment, we generated serial optical sections using confocal microscopy from around 60 to 150 nuclei of neutrophils in both resting and activated states. Image stacks were processed with NEMO software [42] to investigate the nuclear positioning of the p and q telomeres and the distance between them.

#### Position of the p and q telomeres

**Figure 1:**
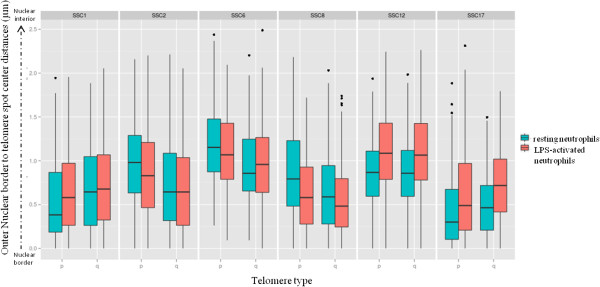
**Position of p and q telomeres towards the outer nuclear border.** Representation in box plots of distances (μm) between nuclear border and telomere spot centres (p and q) in resting (green boxes) and in LPS-activated (red boxes) neutrophils for each chromosome pair. NEMO software was used to measure these 3D distances. The x-axis displays the telomere investigated (p or q) for each chromosome and the y-axis the distance in μm between the outer nuclear border and the centre of the telomeric spot.

We first investigated the position of the p and q telomeres near the nuclear periphery in resting and activated neutrophils by measuring the distance between the centre of the telomeric dots and the outer nuclear membrane clearly delimited by the use of anti-lamin B (Additional file 3: Figure S2). This analysis was performed for the 6 different chromosomes SSC1, SSC2, SSC6, SSC8, SSC12 and SSC17: for 3 chromosomes (SSC2, SSC6, SSC8), the q telomeres were found to be significantly closer to the outer nuclear border compared to the p telomeres both in resting and activated states (Figure 1; Additional file 4: Table S2). For SSC12, no significant difference was found between the mean distances of p and q telomeres from the outer membrane, while for SSC1 and SSC17, the p telomeres were significantly closer to the border than the q telomeres in both states (Figure 1).

We next investigated whether activation of neutrophils modifies the position of telomeres relative to the nuclear outer border. This analysis revealed that the p telomeres change their position significantly when the neutrophils are activated, moving closer to the nuclear outer border for SSC2, SSC6 and SSC8. Inversely, for the largest chromosome (SSC1) and for the two smaller chromosomes (SSC12 and SSC17), the distances between the p telomere and the nuclear outer border increased significantly in the activated state. When the same analysis was performed for the q telomeres, activation was found to have a weaker effect on their positioning, as a change was observed for only three chromosomes (SSC8, SSC12 and SSC17), with the same behaviour as observed for the p telomeres of these chromosomes (Figure 1).

#### Number of nuclei harbouring at least one telomere association

**Figure 2:**
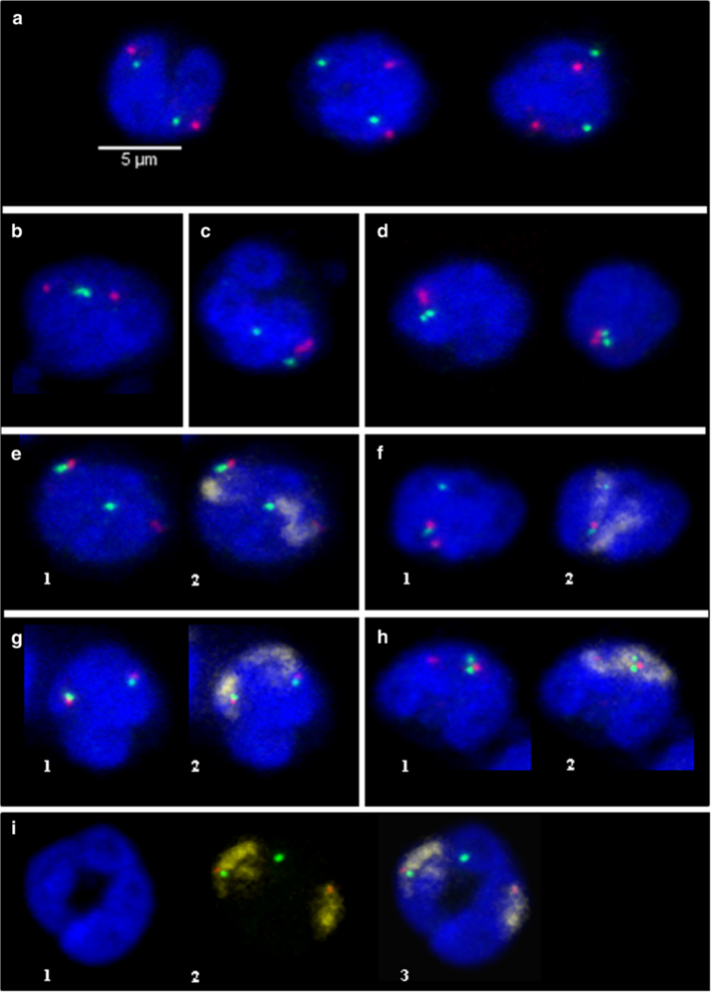
**Representative 3D images displaying examples of telomeric associations.** 3D FISH experiments were carried out with p and q telomeric probes on structurally preserved neutrophil nuclei; maximum intensity projections of confocal image stacks are shown. p telomeres are labelled in red with Alexa 568, q telomeres in green with Alexa 488 and chromosome painting probes in pink with Alexa 633. DNA was counterstained in blue with DAPI. Examples of nuclei with: (**a)** no association, (**b)** qq association, (**c)** pp association, (**d)** pp + qq associations, (**e)** 1- one pqloop association 2- same nucleus after addition of the chromosome painting probe, (**f)** 1- one pqcross association 2- same nucleus after addition of the chromosome painting probe, (**g)** 1- two pqloop associations, 2- same nucleus after addition of the chromosome painting probe, (**h)** 1- one pqloop + one pqcross associations, 2- same nucleus after addition of the chromosome painting probe, (**i)** example of extension of one chromosome territory on two separated lobes: one q telomere is far from its home territory. 1- nucleus, 2- chromosome 1 painting probe combined with p and q subtelomeric probes, 3- merged image.

We first determined for each chromosome pair the percentage of nuclei harbouring at least one association between two telomeres (including the pp, qq or pq associations). This analysis was performed for both states (resting and LPS-activated). Figure 2 provides an example of typical patterns observed in neutrophil nuclei after hybridization with subtelomeric-specific probes. As a comparison of the data obtained in these two states revealed no difference, they were pooled for the following analyses (Table 1). The percentage of nuclei harbouring at least one telomeric association varies significantly (ANOVA, p-value < 2.2 10^-16^) between chromosomes: from 32% for SSC1, SSC6 and SSC17 to 62% for SSC12. However, this percentage can be the same for chromosomes very different both in morphology (SSC1 being submetacentric, SSC6 subtelocentric and SSC17 telocentric) and in size (with SSC1 corresponding to 12.1% of the total porcine genome length and SSC17 only 2.7%). Comparing the data from these chromosomes suggested that there is no correlation between the percentage of nuclei with telomeric associations and chromosome size as confirmed by a regression analysis (Additional file 5: Figure S3).

**Table 1:** Number of neutrophil nuclei harboring at least one telomeric association

**Chromosomes**	**Neutrophil State**	**Number of nuclei with 0 telomeric association**	**Number of nuclei with at least one telomeric association**	**Total number of nuclei scored**	**Effect of activation**	**Pool of data from resting and activated states**	
					***p *value**^**a**^		
						**Number of nuclei with 0 telomeric association**	**Number of nuclei with at least one telomeric association**
		**(%)**	**(%)**				
						**(%)**	
							**(%)**
**SSC1**	R	90 (67%)	45 (33%)	135	0.83	182 (**68%)**	87 (**32**%)
	A	92 (68.7%)	42 (31.3%)	134			
**SSC2**	R	88 (61%)	57 (39%)	145	0.11	196 (**66**%)	103 (**34**%)
	A	108 (70%)	46 (30%)	154			
**SSC6**	R	101 (72%)	39 (28%)	140	0.21	192 (**68**%)	89 (**32**%)
	A	91 (65%)	50 (35%)	141			
**SSC8**	R	89 (65%)	47 (35%)	136	0.66	183 (**67**%)	90 (**33**%)
	A	94 (69%)	43 (31%)	137			
**SSC12**	R	56 (36%)	98 (64%)	154	0.73	109 (**38%**)	181 (**62%**)
	A	53 (39%)	83 (61%)	136			
**SSC13**	A	66 (63%)	38 (37%)	104		66 (**63**%)	38 (**37**%)
**SSC17**	R	106 (71%)	43 (29%)	149	0.25	201 (**68**%)	96 (**32**%)
	A	95 (64%)	53 (36%)	148			

*R* resting state, *A* activated state.

^a^*p* value: test on the effect of the activation process: χ^2^ comparisons of the number of nuclei with 0 or at least 1 telomeric association in resting and LPS-activated neutrophils. Statistical significance *p* ≤0.05.

#### Preferential association types

For each chromosome pair, we then tried to determine which type of telomeric association (pp, qq or pq) is observed between homologous chromosomes when associations between telomeres occur. The use of two different colours to label the p and q telomeres enabled us to define whether we had a pp, qq or pq (Figure 2b-h) association. This analysis was done separately for each chromosome pair in each state (resting and LPS-activated). No difference was observed in the two states analyzed, so the data were pooled (Table 2). The analysis revealed that the pq associations are the most numerous except for SSC13. Indeed, this chromosome behaves differently compared to all other chromosomes tested. Overall, few telomeric associations were detected and these were mainly pp associations. To determine whether the pq telomeric associations observed are the result of an association between the p and q telomeres of one chromosome or by the association of the p and q telomeres of the two homologs, we performed 3D FISH combining the p and q subtelomeric probes and the corresponding whole chromosome paint (Figure 2e-h). This demonstrated that the pq associations result essentially from an association between the p and q telomeres of the same chromosome rather than an association between the p and q telomeres of the two homologs. This intrachromosomal tethering of telomeres that can be called “chromosome bending” occurs at high frequency as it accounts for 60% to 92% of the pq associations observed. This is the case for all the chromosomes investigated regardless of size or morphology (Table 2). This type of association will be denoted pqloop (illustrated in Figure 2e) while the association between the p and q telomeres of the two homologs will be denoted pqcross (illustrated in Figure 2f). This analysis done with the chromosome painting probes combined with the subtelomeric probes revealed that in some cases, one telomere can reside far from its chromosome territory showing that a chromatin loop can extend between the lobes. We carefully analyzed the images in order to quantify this phenomenon (illustrated in Figure 2i). It varied from 25% to 42% depending on the chromosomes (data not shown).

**Table 2:** **Number of pp**, **qq**, **pqloop and pqcross associations observed in neutrophils** (**resting** + **LPS activated**)

**Type of telomeric association**	**SSC1**	**SSC2**	**SSC6**	**SSC8**	**SSC12**	**SSC13**	**SSC17**
	(**n** = **269**)	(**n** = **299**)	(**n** = **281**)	(**n** = **273**)	(**n** = **290**)	(**n** = **104**)	(**n** = **297**)
**pp**	28 (**21**% ^**a**^)	28 (**24**% ^**a**^)	13 (**12% **^**a**^)	17(**16% **^**a**^)	51(**22% **^**a**^)	25(**62% **^**a**^)	26(**22% **^**a**^)
**qq**	27 (**21**% ^**a**^)	15 (**13% **^**a**^)	26 (**25% **^**a**^)	14(**13% **^**a**^)	13(**5% **^**a**^)	10(**25% **^**a**^)	14(**12% **^**a**^)
**pq**	76 (**58%**^**a**^)	73 (**63% **^**a**^)	66 (**63% **^**a**^)	73(**71% **^**a**^)	171(**73%**^**a**^)	5 (**13% **^**a**^)	79(**66% **^**a**^)
***pqloop***	***54***	***64***	***56***	***65***	***145***	***3***	***70***
***pqcross***	(***41%***^***a***^)	(***55***%^***a***^)	(***53***%^***a***^)	(***63***%^***a***^)	(***62***%^***a***^)	(***8***%^***a***^)	(***59% ***^***a***^)
	(***71% ***^***b***^)	(***88***%^***b***^)	(***85***%^***b***^)	(***89***%^***b***^)	(***85***%^***b***^)	(***60***%^***b***^)	(***89% ***^***b***^)
	***22***	***9***	***10***	***8***	***26***	***2***	***9***
	(***17% ***^***a***^)	(***8***%^***a***^)	(***10***%^***a***^)	(***8***%^***a***^)	(***15***%^***a***^)	(***5***%^***a***^)	(***7% ***^***a***^)
	(***29% ***^***b***^)	(***12***%^***b***^)	(***15***%^***b***^)	(***11***%^***b***^)	(***15***%^***b***^)	(***40***%^***b***^)	(***11% ***^***b***^)
**Total**	131(**100%**)	116(**100%**)	105(**100%**)	104(**100%**)	235(**100%**)	40(**100%**)	119(**100%**)

n = total number of nuclei scored (resting + activated states).

^a^percentage of pqloop or pqcross calculated on the total number of associations.

^b^percentage of pqloop or pqcross calculated on the total number of pq associations.

**Figure 3:**
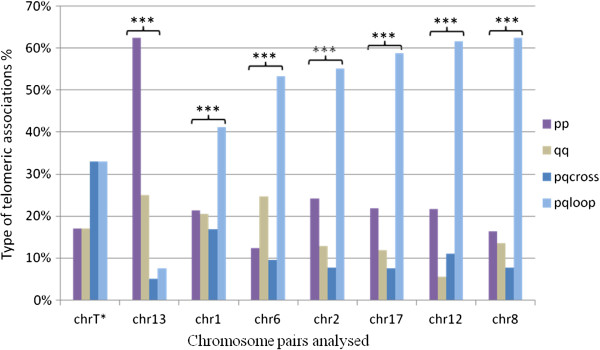
**Distribution of telomeric associations observed between homologs and comparison to the theoretical distribution.** The observed distributions of pp, qq, pqloop and pqcross associations between homologs for the 7 pairs of chromosomes are compared to the theoretical distribution (denoted chrT) calculated under the hypothesis of no preferential association type. The x-axis displays the chromosome pairs and the y-axis the percentage of each type of association observed. Statistical significance is assessed by a χ^2^ test: *p < 0.05, **p < 0.01, ***p < 0.001.

Comparisons revealed that the distribution of pp, qq, pqloop and pqcross associations differs significantly among the 7 chromosomes analyzed (ANOVA *p* < 10^-16^). For each chromosome, we then compared the distribution of pp, qq, pqloop and pqcross associations observed to the theoretical distribution expected if each of the 6 telomeric associations occurs with the same probability and independently, i.e. there is no preferential association type. Taking these data into account, we found that for each chromosome, the observed probabilities of the type of associations differ from the theoretical probabilities (p < 0.001) (Figure 3), showing that the type of telomeric association influences the probability of association.

**Figure 4:**
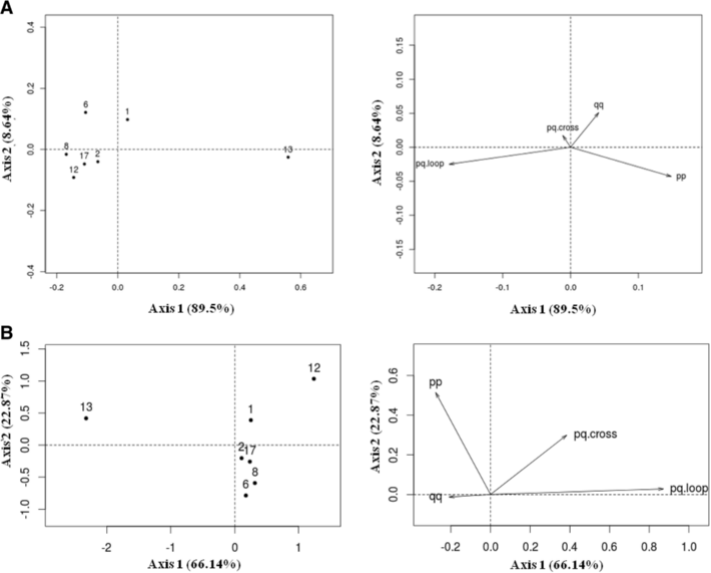
**Principal Component Analysis** (**PCA) graph for telomeric association data.** The two first principal components are plotted with the proportion of variance explained by each component printed next to the axis labels. **A-** Relative frequencies of each association type: the graph clearly shows that most of the variance can be attributed to two types of associations: pp and pqloop; **B**- Probabilities of telomeric associations by type: most of the variance can be attributed to pqloop frequency.

To pursue the investigation on chromosome comparison, a Principal Component Analysis (PCA) was computed on the relative frequencies of each association type i.e. the distribution of association type given an association occurs (Figure 4A). The first axis explains 89.5% of the total observed variance and represents chromosomes based on pqloop and pp association relative frequencies which are negatively correlated. SSC13 has preferentially pp associations and a low frequency of pqloop association while SSC2, SSC8, SSC12 and SSC17 have a low frequency of pp associations and a high frequency of pqloop associations. The second axis explains 8.6% of the total observed variance and represents chromosomes based on qq association relative frequencies. SSC1 and SSC6 have preferentially qq associations. Overall, in terms of telomeric association, SSC13 behaves differently to all the other chromosomes analyzed.

#### Frequencies of telomere associations

For each chromosome pair, we estimated the frequency at which a telomeric association (pp, qq, pqcross or pqloop) occurs by calculating the probabilities of association (see Materials and Methods). A classical nested binomial regression model was used to evaluate the existence of a significant effect of LPS-activation and of telomere association types (pp, qq, pqloop, pqcross). We first compared the association probabilities in resting and activated nuclei (Additional file 6: Table S3). A difference was found only for SSC2 but it remains weak as shown by the p value. We therefore considered that the activation process has no effect on the frequency of telomeric association and the data were pooled. We then sought to see whether these frequencies are the same, irrespective of the type of association. All the chromosomes investigated were found to be significantly association-type dependent (Additional file 6: Table S3). To take our investigation even further and compare the chromosomes with one another, we conducted a principal component analysis. The first axis explains 66% of the total observed variance and discriminates between SSC12 and SSC13 on pqloop association frequency, which is high for SSC12 and low for SSC13. The second axis explains 23% of the total observed variance and opposes, on pp association frequency, SSC12 and SSC13 (high association frequency) to SSC6 and SSC8 (low association frequency) (Figure 4B).

#### Telomere associations between heterologous chromosomes

**Figure 5:**
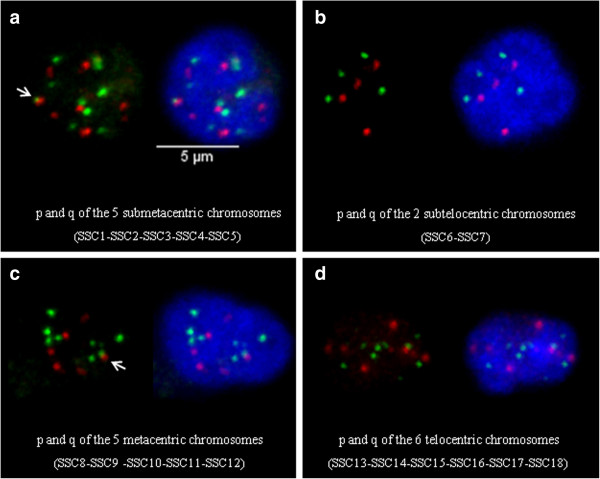
**Representative 3D images displaying examples of telomeric associations between heterologous chromosomes.** 3D FISH with pools of telomeric probes on structurally preserved neutrophil nuclei; maximum intensity projections of confocal image stacks are shown. p telomeres are labelled in red with Alexa 568, q telomeres in green with Alexa 488 and DNA was counterstained in blue with DAPI. Pools of p and q telomeric probes for (**a)** the 5 submetacentric chromosomes (SSC1-SSC2-SSC3-SSC4-SSC5), (**b)** the 2 subtelocentric chromosomes (SSC6-SSC7), (**c)** the 5 metacentric chromosomes (SSC8-SSC9-SSC10-SSC11-SSC12), (**d)** the 6 telocentric chromosomes (SSC13-SSC14-SSC15-SSC16-SSC17-SSC18). White arrows point to pq associations.

**Figure 6:**
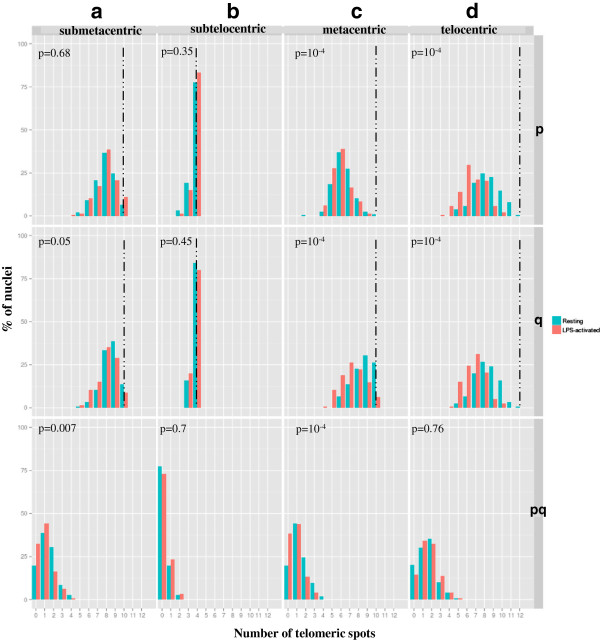
**Analysis of the level of telomere clustering in pools of heterologous chromosomes.** Distribution of the number of telomeric spots (pp, qq, pq) per nucleus in neutrophils (resting in green, LPS-activated in red) using a pool of p, q and p + q telomeric probes labelling the telomeres of: (**a)** the 5 submetacentric chromosomes (SSC1 to SSC5), (**b)** the 2 subtelocentric chromosomes (SSC6 and SSC7), (**c)** the 5 metacentric chromosomes (SSC8 to SSC12), (**d)** the 6 telocentric chromosomes (SSC13 to SSC18). The x-axis displays the number of telomeric spots counted and the y-axis the % of nuclei harbouring the numbers of spots concerned. The dashed lines indicate the expected number of spots according to the number of telomeres labelled in the pool. The P values show comparisons between the two states (resting and activated) using a χ^2^ test.

To complete the analysis of telomeres from homologs, we tried to determine whether the telomeres of heterologous chromosomes associate. For this, we chose to investigate the behaviour of p and q telomeres in pools of chromosomes, composed of chromosomes belonging to the same group according to their morphology (the analysis of all the possible combinations between heterologous chromosomes would have required too many experiments). Four different pools were analyzed: **(1)** the 5 submetacentric chromosomes: SSC1 to SSC5, **(2)** the 2 subtelocentric chromosomes: SSC6 and SSC7, **(3)** the 5 metacentric chromosomes: SSC8 to SSC12 and **(4**) the 6 telocentric chromosomes: SSC13 to SSC18. For each pool, two probes were generated containing either the p subtelomeric or the q subtelomeric probes (Additional file 2: Figures S1h-k). These probes were labelled in red and green respectively and hybridized on 3D-preserved resting and LPS-activated neutrophil nuclei (Figure 5). More than 140 nuclei were analyzed for each pool in each condition. For all the pools analyzed, the average number of telomere signals was inferior to the expected number both for p and q telomeres, suggesting that clustering occurs. Although the level of clustering remains globally low irrespective of the pool analyzed (Additional file 7: Table S4), it is higher in the metacentric and telocentric groups for p and q telomeres compared to the submetacentric and subtelocentric groups. In addition, in the metacentric group, the p telomeres associate significantly more than the q telomeres, both in resting and activated states. We then investigated whether the activation process modifies this level of association. When the neutrophils are activated, the tendency of the p telomeres to associate in the metacentric and telocentric groups increases significantly (Figure 6). The same phenomenon is observed for the q telomeres. We also looked for pq associations between telomeres in pools containing heterologous chromosomes. Only a very small number of this type of association was detected (Figure 5a-c) (Additional file 7: Table S4). In addition, in the submetacentric and metacentric groups, this number decreases significantly in LPS-activated neutrophils.

## Discussion

### Positioning the p and q telomeres toward the outer nuclear border

Telomeres play an essential role in preserving chromosome integrity and by their contribution to chromosome positioning within the nucleus. Their organization has been studied in various cell types. In yeast, telomeres form limited clusters that appear to be attached to the nuclear envelope while in humans, telomeres are dispersed throughout the nucleus. However some telomeres show a preferential association with the nuclear periphery. This is the case for the q telomere of human chromosome 4 [43]. Similarly a preferential association with the nucleolus has been shown for the short arms of acrocentric chromosomes due to the presence in these arms of rDNA clusters [44]. Telomeres were reported to be strongly tethered to the nuclear architecture by lamins, the main component of the lamina layer forming the interface between chromatin and the inner nuclear membrane. However, in spite of their close association with the nuclear lamina, telomeres are highly mobile structures, as shown by live imaging studies [45]. While the organization of telomeres has been studied in various cell types, information available for neutrophils is very limited, probably due to the complex structure of their nuclei. We studied the position of p and q telomeres of different chromosomes in relation to the outer nuclear border which, in these cells with polylobed nuclei, corresponds to the cell border directly in contact with the cytoplasm. Our data demonstrate that for SSC2, SSC6, SSC8 and SSC13, the q telomeres were significantly closer to the nuclear outer border compared to the p telomeres, while this was not the case for the two smaller chromosomes SSC12 and SSC17. However no correlation with size or chromosome morphology can be made as SSC1, the largest chromosome, behaves like the two smaller chromosomes. Upon neutrophil activation by LPS, the p telomeres move significantly towards the nuclear border while this effect is less pronounced for the q telomeres. Our data are consistent with the observations on living cells, showing that telomeres are highly mobile structures and that the motion of chromosome ends is highly heterogeneous [45]. Short telomeres have been shown to be more mobile than longer telomeres. This greater motility could be the result of the active decompaction of a large sub-telomeric region.

### Defining the type of association between the subtelomeres of homologous chromosomes

Telomere associations have been found to be more prevalent in the interphase of non-cycling cells than in their cycling counterparts [9]. Different roles have been suggested for these associations, including maintenance of chromosome positional stability and creation of a 3D transcription-permissive environment [46]. In neutrophils, which are terminally differentiated non-cycling cells, but transcriptionally active, we also found associations between the subtelomeres of homologous chromosomes. A high frequency of association between the subtelomeres of the short and the long arms (pq association) irrespective of the chromosomes studied, was described in non-cycling fibroblasts [12]. In neutrophils, our data demonstrate a more variable pattern of association depending on the chromosomes (Figure 4). We also found that when an association occurs, a pq association is more prevalent (representing 58% to 73% of the observed associations) except for SSC13 for which this type of association represents only 13% of all associations. For this chromosome, pp-telomere tethering is more prevalent. The same observation was made for human chromosome 19 in non-cycling lymphocytes, where qq associations were found in up to 80% of all nuclei studied [10].

The use of whole chromosome specific paints enabled us to clearly determine that when pq associations occur, they mainly originate from intrachromosomal telomeric tethering between homologs denoted pqloop, the pqcross associations remaining marginal (Table 2).

There are clear similarities between the chromosome bending seen in these non-cycling cells and that observed in spermatozoa, which are also terminally differentiated cells [15]. In spermatozoa, telomeres associate in doublets of telomeres from the same chromosome. It has been hypothesized that this chromosome looping is a consequence of the associations between specific subtelomeric DNA sequences, protein complexes and chromatin structures located in the terminal domains of chromosome arms [15]. These associations are probably more complex than previously through as two hundred and ten proteins have been shown to interact with DNA and might influence telomeric structure [47]. In this context, chromosome looping would play an important functional role by promoting telomere-telomere interactions in specialized domains. Our data are in agreement with these findings as we showed that the percentage of pqloop is high for some chromosomes (representing 63% and 62% of all associations for SSC8 and SSC12 respectively) and low for others (8% for SSC13).

Our results demonstrate that the LPS-activation process does not change the pattern of telomeric associations. A similar observation was made concerning human lymphocytes: after stimulation by a mitogen, only minor changes in the incidence of telomere associations were reported [10]. However, our data show that LPS activation induces significant changes in the position of telomeres in relation to the outer nuclear border.

### Defining the probability of telomeric association in a pair of chromosomes

We first analyzed what types of association occur between the subtelomeres of homologous chromosomes. The second level of analysis consisted in determining at what frequency these associations occur. The analysis revealed that: (**1)** the probability of an association between the subtelomeres of homologous chromosomes differs significantly between chromosomes. Two chromosomes (SSC12 and SSC13) behave differently compared to the others: they each show a high probability that their p telomeres associate. In addition, SSC12 also has the highest probability of intrachromosomal associations occurring between the p and q telomeres, while this event remains very rare for SSC13, (**2)** the probabilities of the different types of association occurring can differ significantly for a given chromosome pair: for example, the probability of an association between the two p subtelomeres of SSC13 is high while the probability of a pq association (pqloop or pq cross) is very low (Figure 4B).

Overall, therefore, the probability of a telomeric association is high for SSC12. It has been shown that associations of telomeres in interphase nuclei might contribute to the establishment of transcription-permissive 3D nuclear compartments [46]. This could be an explanation, as gene density is higher in this porcine chromosome than in the other chromosomes investigated [48], [http://www.ensembl.org/Sus_scrofa/Location/Genome]. SSC17, which is similar in size (69 Mb compared to 63 Mb for SSC12) but very different in gene density (8.4 and 15.9 genes/Mb for SSC17 and SSC12 respectively), behaves differently in terms of telomeric associations, which supports this hypothesis. Our results concur with the observation made recently by Foster and collaborators [49] on the non-random organization of the porcine genome in the nucleus of cells from various origins or lineages.

### Analyzing the telomere associations between heterologous chromosomes

Little information is available on the association of telomeres from heterologous chromosomes. This phenomenon was studied in human lymphocytes using specific probes for the p and q arms of chromosomes 8, 9 and 19. No significant incidence of telomere associations was found between these chromosomes [10]. However, no information is available for neutrophils. Using pools containing the p and q telomeres of different chromosomes, we defined the level of telomere associations within the 4 groups of chromosomes tested (submetacentric, subtelocentric, metacentric and telocentric). The analysis revealed that the level of telomere associations differs between the groups: the lowest level is observed for the subtelocentric chromosomes. Overall, it remains fairly small, with the ratio (number of expected dots)/(number of observed dots) ranging from 1.2 to 1.8. This suggests that the higher level of clustering observed with the PNA probe (ratio of 4) is probably the result of telomeric associations occurring between chromosomes belonging to different groups.

## Conclusions

This study adds new data on genome organization in one cell type which is relatively complex due to the polylobed structure of its nucleus and which is particularly important as it plays a major role in immune response. We focussed our study on 3D telomere organization and its modifications when these immune cells are activated in response to a pathogen aggression. We showed that (**1)** for all chromosomes, the p telomeres changed their position significantly during the activation process while the effect is less pronounced for the q telomeres; (**2)** the percentage of nuclei harbouring at least one telomeric association varies significantly between chromosomes, the smallest metacentric chromosome SSC12, which is also the richest in gene-density, harbouring a high level of association between its telomeres; (**3)** the distribution of types of telomeric associations differs significantly between the 7 chromosomes, the largest telocentric chromosome SSC13 showing a very different pattern compared to the other chromosomes; (**4)** the frequency of telomeric association occurring between homologs is highly dependent on the type of association and the type of chromosome; (**5)** overall, the LPS-activation process induces only minor changes in these patterns of associations, probably because the drastic structural changes come later during NETosis; and finally the most noteworthy (**6)** when telomeric associations occur between homologs, the most prevalent association type consists of pq tethering of the telomeres from one chromosome, suggesting that chromosomes bend back on themselves in neutrophils to promote telomere-telomere interactions in specialized domains.

## Methods

### Ethics statement

Our experiment was conducted in accordance with the French national regulations for human care and use of animals in research. Blood samples were collected on large-white pigs from UMR INRA Toxalim under the experimentation agreement number TOXCOM/0020/PP IO for the collection of blood samples.

### Neutrophil isolation and activation

Neutrophils were isolated from freshly drawn venous blood of 7 to 9 month-old healthy pigs as described previously [33]. Briefly, after density gradient centrifugation (1.077 g/l, Lymphoprep, Eurobio), the pellet containing the neutrophils was submitted to a lysis solution (NH_4_Cl 0.15 M – KHCO_3_ 0.01 M EDTA Na_2_ 1 μM) at room temperature for 10 min to eliminate the red cells. The cell suspension was then centrifuged at 300 g for 20 min and the pellet was resuspended in 1 ml of RPMI 1640. Purity of cell population was estimated at 90% by microscopic examination of May-Grünwald Giemsa (Sigma-Aldrich) stained blood smears and cell viability was found to be greater than 95% by a Trypan blue dye exclusion test. The neutrophil sample was divided in two: one part was directly used for spreading on slides (resting neutrophils) and the other part (adjusted at 7 × 10^6^ cells/mL) was incubated by shaking with LPS (10 μg/mL for 3 h at 37°C) to produce the LPS-stimulated batch. The cells were spread on slides as described previously [33].

### DNA probes

Subtelomeric probes were selected using recent data from the pig genome sequencing project (http://pre.ensembl.org/Sus_scrofa_map/Location/Genome). Each probe consisted of: (**1)** 2 BACs specific to the p or q telomeric regions of a chromosome pair, (**2)** 4 different pools of BACs specific to the p or q telomeric regions of submetacentric chromosomes (SSC1 to SSC5), subtelocentric chromosomes (SSC6 and SSC7), metacentric chromosomes (SSC8 to SSC12) and finally telocentric chromosomes (SSC13 to SSC18). These BAC clones were obtained from the Biological Resources Centre – GADIE (http://www.crb-gadie.inra.fr). All the BACs used in this study were presented in Additional file 1: Table S1. For each chromosome, p (probe 1) and q (probe 2) telomeric BACs were combined. Probes 1 and 2 were previously labelled by random priming by incorporation of dUTP Alexa 568 and dUTP Alexa 488 respectively, using the Bioprime DNA labelling kit (Invitrogen). Similarly, the 4 pools of p and q telomeric probes were labelled by incorporation of dUTP Alexa 568 and dUTP Alexa 488 respectively. Products from the reaction were precipitated with porcine Cot-1 DNA (Applied Genetics Laboratories, Melbourne, USA) and salmon sperm DNA (Eurobio). Each probe was dropped onto slides at a final concentration of 100 ng/μL in a hybridization buffer (Sigma-Aldrich).

Chromosome specific painting probes from flow-sorted chromosomes [50] were individually labelled by random priming with Biotin (Invitrogen) and combined with the appropriate subtelomeric probes. Biotin was revealed by streptavidin conjugated to Alexa 633 (Invitrogen). The specificity of all probes was tested by 2D FISH on porcine metaphases prepared from lymphocytes according to protocols which had previously been described [51] (Additional file 2: Figures S1a-g).

### 3D-FISH experiments

3D FISH experiments were carried out according to [33]. Briefly, after permeabilization of the neutrophils and RNAse A treatment, cells and probes were simultaneously heat-denatured at 75°C for 8 min and then incubated for 72 h at 37°C. The entire procedure was carried out in a DAKO hybridizer. Post-hybridization washes were performed first in 2 × SSC at room temperature (twice) for 15 min, then three times for 15 min each in 2 × SSC 50% formamide pH 7.0 at 45°C, and finally three times for 15 min each in 0.1 × SSC at 50°C. Nuclei were counterstained with 4',6'diamidino-2-phenylindole (DAPI) in Vectashield medium (Vector Laboratories). For the visualization of the nuclear membrane, the samples were incubated with an anti-lamin B antibody (Santa Cruz Biotechnology) at 1:50 dilution in PBS containing 2% serum albumin for 1 h at room temperature and incubated with an anti-goat Alexa 488 secondary antibody (Invitrogen).

### Confocal microscopy and image analyses

Confocal microscopy was carried out using a Leica TCS SP2 confocal microscope (Leica Instruments) equipped with an oil immersion objective (plan achromatic 63× N.A. = 1.4). The Z-stacks were acquired at 1024 × 1024 pixels per frame using an 8-bit pixel depth for each channel at a constant voxel size of 0.093 × 0.093 × 0.244 μm. Typically, a stack of 45 confocal planes was acquired. Segmentations and 3D measurements between objects (3D distances between nucleus outer border and subtelomeric probe centres, or between subtelomeric probe centres) were performed using NEMO software [42]. The nucleus segmentation was validated by labelling the nuclear membrane with anti-lamin B (Additional file 3: Figure S2) and more generally, the segmentation for each cell (nucleus and objects) was validated by manual comparisons with the raw images.

### Telomere associations

Telomeres were defined as associated if the 3D distance between their spot centres was inferior to 2 × r, r being the spot radius. We determined for each chromosome pair how many nuclei were in each conformation (denoted C1 to C14 – Additional file 8: Figure S4a). Then we calculated the number of associations and non-association events for each type of telomeric association considering that there are 6 possibilities (1 pp, 1 qq, 2 pqloop and 2 pqcross) (Additional file 8: Figure S4b).

### Statistical analyses

#### Distance of the p and q telomeres from the nuclear outer border

Pairwise comparisons of the average values (border-to-border distances between the spot and the outer nuclear periphery) over the two cell populations (resting and activated neutrophils) were carried out using the Mann–Whitney Wilcoxon test R software, [52]. Similarly in each state, the positions of the p and q telomeres were compared using the same statistical test.

#### Test on random association between p and q telomeres

For each chromosome, the distribution of pp, qq, pqloop and pqcross associations observed was compared to the theoretical distribution (*p*) expected if these associations occur at random and independently (*p* (pp or qq) = 1/6; *p* (pqloop or pqcross) = 1/3) using a χ^2^ test. *p* values <0.05 were considered as significant.

#### Frequency of telomeric associations

To determine the frequency at which a telomeric association occurs, we estimated a probability of association for each type (pp, qq, pq_loop_ or pq_cross_) as follows:

Probability of pp association *p*_*pp*_ :

*p*_*pp*_ = Number of pp associations **/** (Number of pp associations + Number of 0 pp association)

and similarly for *p*_*qq*_, *p*_*pqloop*_ and *p*_*pqcross*_ (Additional file 8: Figure S4a).

This estimation derives from the following binomial model. With X_c,t,s,i_ denoting the number of telomeric associations observed in the nucleus (i), in the state (s) (activated/not activated), of the type t (*pp*, *qq*, *pqloop*, *pqcross*) and the chromosome c, and N_c,t,s,i_ the associated maximum number of such associations, we stated that X_c,t,s,i_ follows a binomial distribution: X_c,t,s,i_ ~ Bin(N_c,t,s,i_ , p_c,t,s,i_ ). In order to estimate and test the existence of an effect of different factors such as LPS-activation, chromosomal association level and association type, we fitted the binomial regressions with the following covariates (X stands for interaction) and applied a step by step procedure:

Model 1: chromosome + type + state + chromosome X type + chromosome X state X type

Model 2: chromosome + type + state + chromosome X type

Model 3: chromosome + type

Model 4: chromosome + state

Model 5: chromosome

Model 6: constant

The fit of these nested models was compared using a χ^2^ test.

As a complement, we conducted a Principal Component Analysis on the observed relative frequencies of the number of telomeric associations observed, sorted by type.

## Abbreviations

ANOVA: Analysis of variance; BAC: Bacterial artificial chromosome; CT: Chromosome territory; DAPI: 4',6’Diamidino-2-phenylindole; 2D FISH: Two-dimensional fluorescence in situ hybridization; 3D FISH: Three-dimensional fluorescence in situ hybridization; LPS: Lipopolysaccharide; NET: Neutrophil Extracellular Traps; PNA: Peptide nucleic acid; SSC: Sus scrofa domestica

## Competing interests

The authors declare that there are no conflicts of interest.

## Authors’ contributions

FM carried out all the experiments, protocol design, confocal microscopy, 3D image and data analyses and helped draft the manuscript, DR participated in data analysis and performed the statistical analysis, CD participated in the experiments, MYB designed and developed the project, analysed the data and wrote the manuscript. All authors have read and approved the final manuscript.

## Supplementary Material

Additional file 1: Table S1 List of BAC clones selected to specifically label the p and q telomeres of each porcine chromosome. Click here for file

Additional file 2: Figure S1 Specificity controls of probes by 2D-FISH on porcine metaphases. Click here for file

Additional file 3: Figure S2 Labelling of the nuclear border with anti-lamin B. Click here for file

Additional file 4: Table S2 3D distances between nuclear outer border and telomeric (p/q) spot centres in resting and LPS-activated neutrophils. Click here for file

Additional file 5: Figure S3 Analysis of the correlation between chromosome size (in percentage of total genome length) and percentage of nuclei harbouring at least one telomeric association. Click here for file

Additional file 6: Table S3 Probabilities of telomeric associations. Click here for file

Additional file 7: Table S4 Analysis of telomeric associations in pool of heterologous chromosomes. Click here for file

Additional file 8: Figure S4 Estimation of the number of events (telomeric associations) that can occur in a neutrophil nucleus. Click here for file
