# Supplementary material for: 3D organization of telomeres in porcine neutrophils and analysis of LPS-activation effect
Source: BMC Cell Biol. 2013 Jun 26;14:30. doi: 10.1186/1471-2121-14-30 (PMC3701612; doi:10.1186/1471-2121-14-30)
Supplement: Additional file 1: Table S1 — List of BAC clones selected to specifically label the p and q telomeres of each porcine chromosome. [file 1471-2121-14-30-S1.docx]

**Additional file 1: Table S1 - List of BAC clones selected to specifically label the p and q telomeres of each porcine chromosome.**

| Chromosome | telomere | BAC clones^1^ |
| --- | --- | --- |
| **SSC1** | pter  qter | 428G6  670B11 |
| **SSC2** | pter  qter | 370D12  3B4 |
| **SSC3** | pter  qter | 130B10  639G2 |
| **SSC4** | pter  qter | 100D4  330C8 |
| **SSC5** | pter  qter | 758H12  830B4 |
| **SSC6** | pter  qter | 624C3  855G3 |
| **SSC7** | pter  qter | 445F2  708D3 |
| **SSC8** | pter  qter | 205F3  277F7 |
| **SSC9** | pter  qter | 736D9  564B6 |
| **SSC10** | pter  qter | 483H10  404B11 |
| **SSC11** | pter  qter | 854A11  736F8 |
| **SSC12** | pter  qter | 800G3  851E5 |
| **SSC13** | pter  qter | 829B7  39F7 |
| **SSC14** | pter  qter | 90C4  413G8 |
| **SSC15** | pter  qter | 514C10  899B10 |
| **SSC16** | pter  qter | 172B11  230H12 |
| **SSC17** | pter  qter | 694C9  435B10 |
| **SSC18** | pter  qter | 485H3  706D7 |

^1^The BAC clones were isolated from the INRA BAC library available at the Biological Resources center GADIE (http://www.crb.jouy.inra.fr)
