# Supplementary figures and images for: 3D organization of telomeres in porcine neutrophils and analysis of LPS-activation effect
Source: BMC Cell Biol. 2013 Jun 26;14:30. doi: 10.1186/1471-2121-14-30 (PMC3701612; doi:10.1186/1471-2121-14-30)

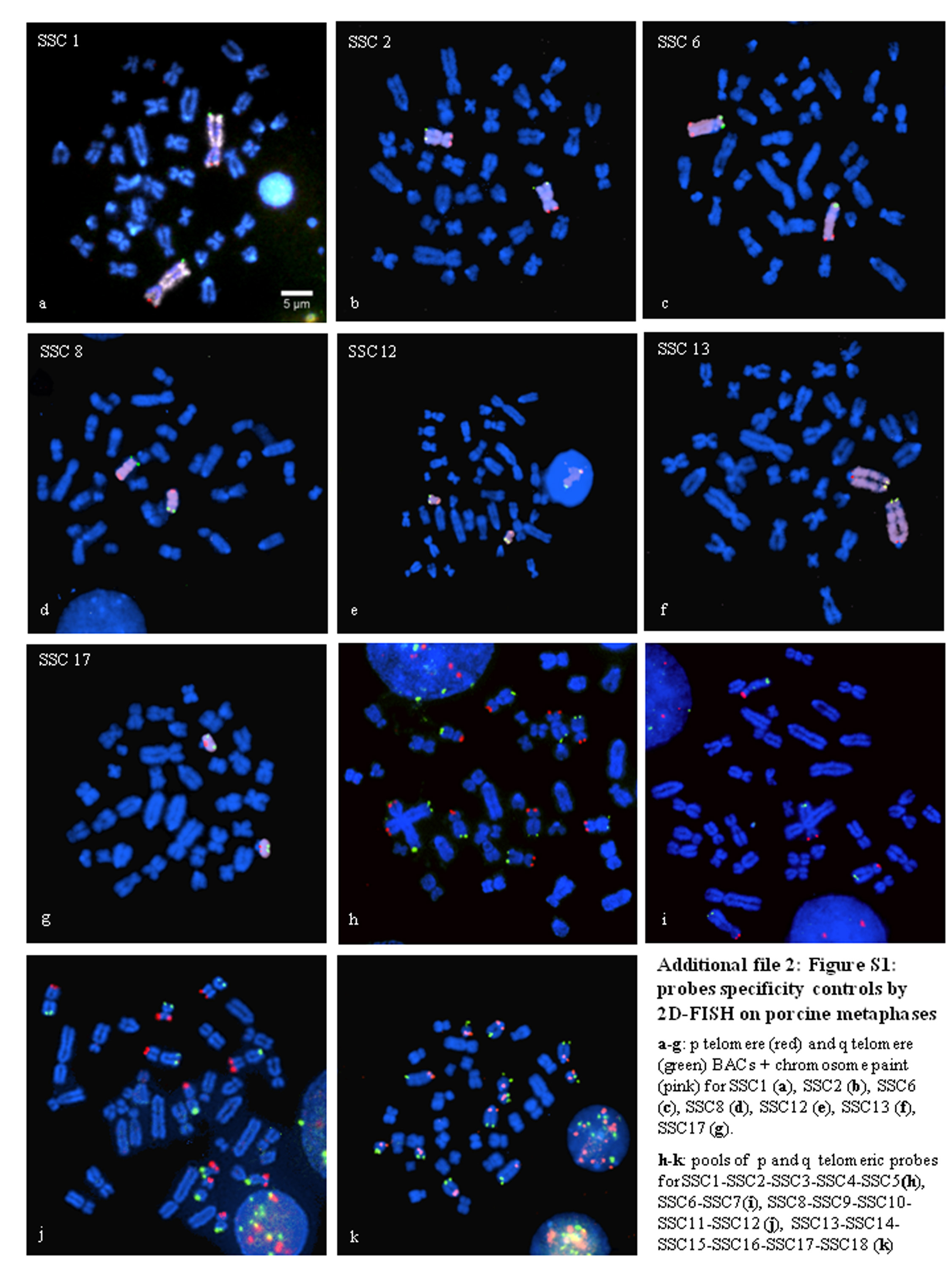

Supplement: Additional file 2: Figure S1 — Specificity controls of probes by 2D-FISH on porcine metaphases. [file 1471-2121-14-30-S2.jpeg]

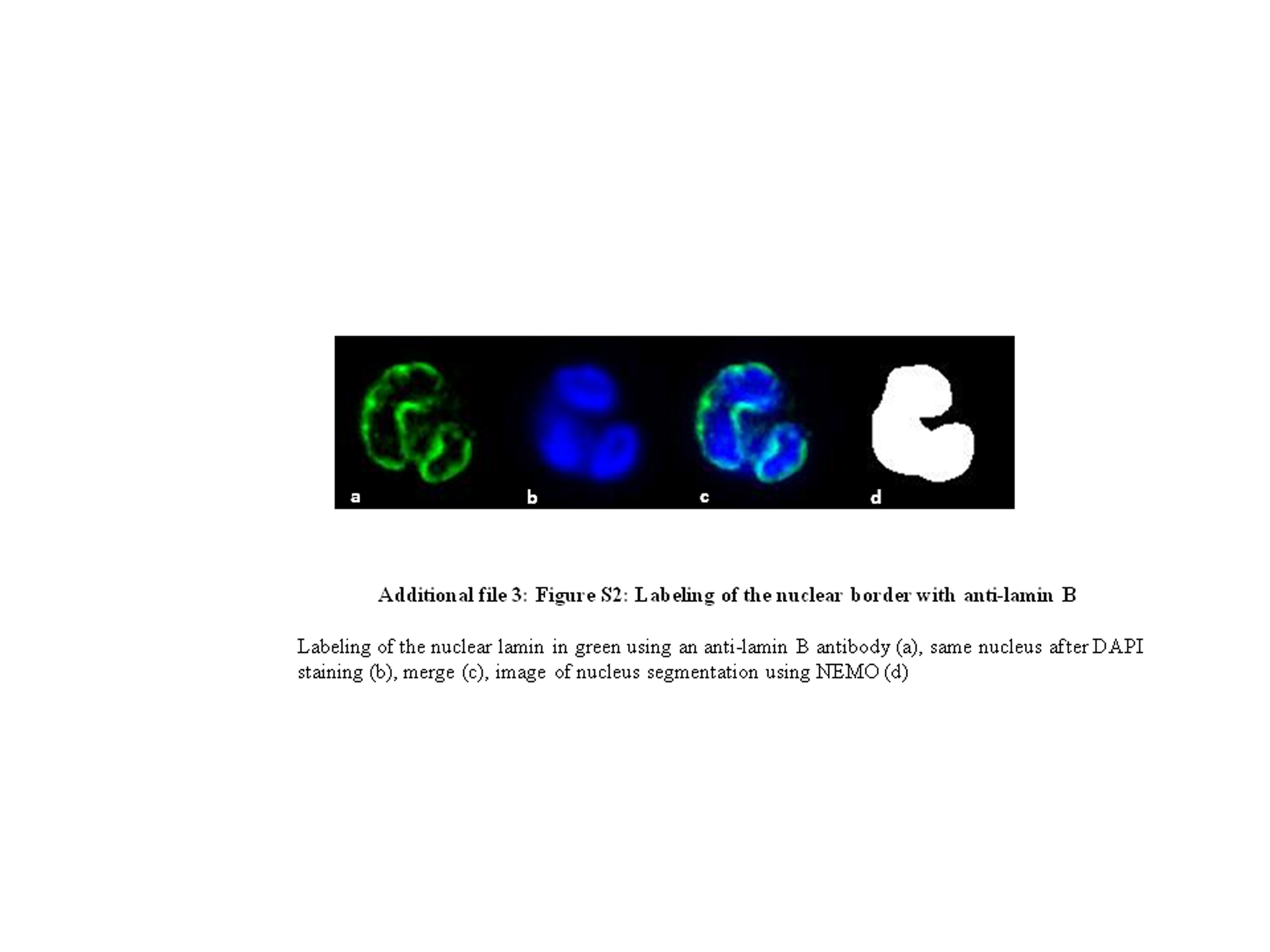

Supplement: Additional file 3: Figure S2 — Labelling of the nuclear border with anti-lamin B. [file 1471-2121-14-30-S3.jpeg]
