# Supplementary material for: 3D organization of telomeres in porcine neutrophils and analysis of LPS-activation effect
Source: BMC Cell Biol. 2013 Jun 26;14:30. doi: 10.1186/1471-2121-14-30 (PMC3701612; doi:10.1186/1471-2121-14-30)
Supplement: Additional file 4: Table S2 — 3D distances between nuclear outer border and telomeric (p/q) spot centres in resting and LPS-activated neutrophils. [file 1471-2121-14-30-S4.docx]

**Additional file 4: Table S2 - 3D distances between nuclear outer border and telomeric (p/q) spot centers in resting and LPS-activated neutrophils**

| **Chromosome** | **SSC1** | | | **SSC2** | | | **SSC6** | | | **SSC8** | | | **SSC12** | | | **SSC17** | | |
| --- | --- | --- | --- | --- | --- | --- | --- | --- | --- | --- | --- | --- | --- | --- | --- | --- | --- | --- |
|  | **R**  n=141 | **A**  n=143 | *p value^a^* | **R**  n=149 | **A**  n=155 | *p value^a^* | **R**  n=142 | **A**  n=145 | *p value^a^* | **R**  n=137 | **A**  n=141 | *p value^a^* | **R**  n=161 | **A**  n=142 | *p*  *value^a^* | **R**  n=153 | **A**  n=154 | *p* *value^a^* |
| Dborder-**p**  mean value (µm) | **0.52** | **0.64** | *7.10^-4^* | **0.97** | **0.87** | *0.005* | **1.18** | **1.10** | *0.04* | **0.86** | **0.63** | *6.10^-8^* | **0.86** | **1.11** | *10^-11^* | **0.43** | **0.63** | *10^-5^* |
| Dborder-**q**  mean value (µm) | **0.69** | **0.73** | *0.38* | **0.74** | **0.69** | *0.30* | **0.95** | **0.97** | *0.43* | **0.66** | **0.54** | *0.005* | **0.86** | **1.11** | *10^-10^* | **0.49** | **0.73** | *10^-12^* |
| *p value^b^* | *2. 10^-5^* | *0.03* |  | *2. 10^-9^* | *3 10^-5^* |  | *10^-10^* | *10^-4^* |  | *4 10^-6^* | *0.01* |  | *0.95* | *0.73* |  | *0.007* | *2 10^-4^* |  |

R= resting state; A= activated state; n= number of nuclei analyzed

Dborder-p (q) = distance between nuclei outer border and telomere p (q) spot center

Pairwise comparisons using Mann-Whitney Wilcoxon test:
- *p value^a^* for the comparison of the Dborder-**p** or Dborder-**q** mean values in resting and activated states

*- p value^b^* for the comparison of Dborder-**p** and Dborder-**q** mean values in each state (resting or activated)
