# Supplementary material for: 3D organization of telomeres in porcine neutrophils and analysis of LPS-activation effect
Source: BMC Cell Biol. 2013 Jun 26;14:30. doi: 10.1186/1471-2121-14-30 (PMC3701612; doi:10.1186/1471-2121-14-30)
Supplement: Additional file 5: Figure S3 — Analysis of the correlation between chromosome size (in percentage of total genome length) and percentage of nuclei harbouring at least one telomeric association. [file 1471-2121-14-30-S5.docx]

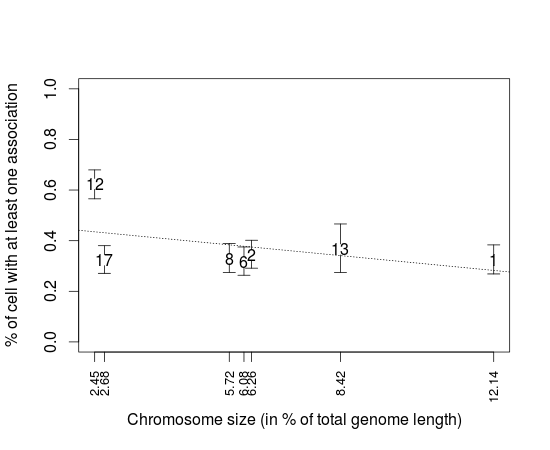


**Additional file 5: Figure S3**

Analysis of the correlation between chromosome size (in percentage of total genome length) and percentage of nuclei harbouring at least one telomeric association.
