# Supplementary material for: 3D organization of telomeres in porcine neutrophils and analysis of LPS-activation effect
Source: BMC Cell Biol. 2013 Jun 26;14:30. doi: 10.1186/1471-2121-14-30 (PMC3701612; doi:10.1186/1471-2121-14-30)
Supplement: Additional file 6: Table S3 — Probabilities of telomeric associations. [file 1471-2121-14-30-S6.docx]

**Additional file 6: Table S3 - Probabilities of telomeric associations**

| Probabilities of telomeric  associations | **SSC1** | | |  | **SSC2** | | |  | **SSC6** | | |  | **SSC8** | | |  | **SSC12** | | |  | **SSC13** | |  | **SSC17** | | |
| --- | --- | --- | --- | --- | --- | --- | --- | --- | --- | --- | --- | --- | --- | --- | --- | --- | --- | --- | --- | --- | --- | --- | --- | --- | --- | --- |
|  | R | A | *p value^b^* |  | R | A | *p value^b^* |  | R | A | *p*  *value^b^* |  | R | A | *p value^b^* |  | R | A | *p value^b^* |  | A | *p value^b^* |  | R | A | *p value^b^* |
| **Association pp** | **0.10** | **0.10** | ***<10^-4^*** |  | **0.08** | **0.10** | ***<10^-12^*** |  | **0.04** | **0.05** | ***<10^-9^*** |  | **0.07** | **0.05** | ***<10^-12^*** |  | **0.19** | **0.15** | ***<10^-16^*** |  | **0.24** | ***<10^-13^*** |  | **0.07** | **0.11** | ***<10^-13^*** |
| **Association qq** | **0.09** | **0.11** |  |  | **0.06** | **0.05** |  |  | **0.09** | **0.10** |  |  | **0.05** | **0.05** |  |  | **0.05** | **0.04** |  |  | **0.10** |  |  | **0.03** | **0.06** |  |
| **Association pqloop** | **0.10** | **0.10** |  |  | **0.14** | **0.07** |  |  | **0.08** | **0.12** |  |  | **0.11** | **0.13** |  |  | **0.25** | **0.25** |  |  | **0.01** |  |  | **0.11** | **0.12** |  |
| **Association pqcross** | **0.04** | **0.04** |  |  | **0.02** | **0.01** |  |  | **0.03** | **0.01** |  |  | **0.01** | **0.02** |  |  | **0.05** | **0.04** |  |  | **0.01** |  |  | **0.02** | **0.01** |  |
| *p value^a^* | *0.89* | |  |  | *0.04* | |  |  | *0.38* | |  |  | *0.56* | |  |  | *0.29* | |  |  |  | |  | *0.27* | |  |

R= resting state; A= activated state

Statistical differences on the probabilities of telomeric associations in resting and activated neutrophils were assessed by binomial regression models (see Methods). Tests were carried out on the existence of: **1)** a LPS-activation effect (p value^a^), **2)** an effect of the type of telomeric associations (pp, qq, pqloop, pqcross) (p value^b^). In this case, the test was done on pooled data (from resting and activated neutrophils) as no effect of LPS-activation was detected.
