# Supplementary material for: 3D organization of telomeres in porcine neutrophils and analysis of LPS-activation effect
Source: BMC Cell Biol. 2013 Jun 26;14:30. doi: 10.1186/1471-2121-14-30 (PMC3701612; doi:10.1186/1471-2121-14-30)
Supplement: Additional file 7: Table S4 — Analysis of telomeric associations in pool of heterologous chromosomes. [file 1471-2121-14-30-S7.docx]

**Additional file 7: Table S4 - Analysis of telomeric associations in pool of heterologous chromosomes**

| **Pool of p and q telomeres** | **Submetacentric**  **(**SSC1-SSC2-SSC3-SSC4-SSC5**)** | | **Subtelocentric**  (SSC6-SSC7) | | **Metacentric**  (SSC8-SSC9-SSC10-SSC11-SSC12) | | **Telocentric**  (SSC13-SSC14-SSC15-SSC16-SSC17-SSC18) | |
| --- | --- | --- | --- | --- | --- | --- | --- | --- |
| **Number of expected spots** | 10 p - 10 q | | 4 p - 4 q | | 10 p - 10 q | | 12 p - 12 q | |
|  | **R**  n=153 | **A**  n=145 | **R**  n=156 | **A**  n=145 | **R**  n=167 | **A**  n=143 | **R**  n=150 | **A**  n=151 |
| Mean number of p spots observed | **7.9** | **8.0** | **3.7** | **3.8** | **6.3** | **5.9** | **8.4** | **6.6** |
| Mean number of q spots observed | **8.5** | **8.0** | **3.8** | **3.8** | **8.6** | **7.3** | **8.2** | **6.8** |
| *p value* | *0.002* | *0.74* | *0.056* | *0.20* | *10^-4^* | *10^-4^* | *0.85* | *0.11* |
| **Mean number of pq spots observed** | **1.4** | **1** | **0.25** | **0.3** | **1.3** | **0.8** | **1.5** | **1.6** |

R= resting state; A= activated state; n= number of nuclei analyzed

Statistical test used: Pearson's Chi-squared test with simulated p-value for the comparison of the number of observed **p** and **q** spots in each state (resting or activated). Statistical significance *p*<0.05
