# Supplementary material for: 3D organization of telomeres in porcine neutrophils and analysis of LPS-activation effect
Source: BMC Cell Biol. 2013 Jun 26;14:30. doi: 10.1186/1471-2121-14-30 (PMC3701612; doi:10.1186/1471-2121-14-30)
Supplement: Additional file 8: Figure S4 — Estimation of the number of events (telomeric associations) that can occur in a neutrophil nucleus. [file 1471-2121-14-30-S8.docx]

**A - Different conformations observed for telomeric associations**

**
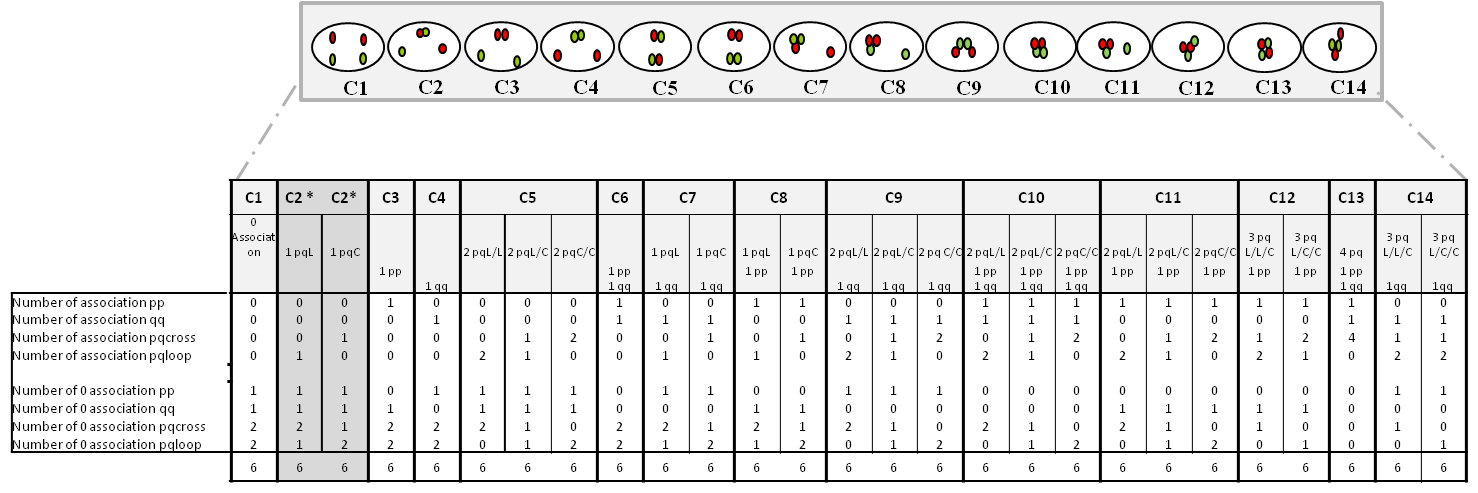
**As an example, one conformation is detailled: C2*: contains one pq association that can result either from an association between the p and q telomeres of the same chromosome (pqloop) or from an association between the p telomere of one chromosome and the q telomere of its homolog (pqcross).

**B – Potential events of telomeric associations in a nucleus**

**
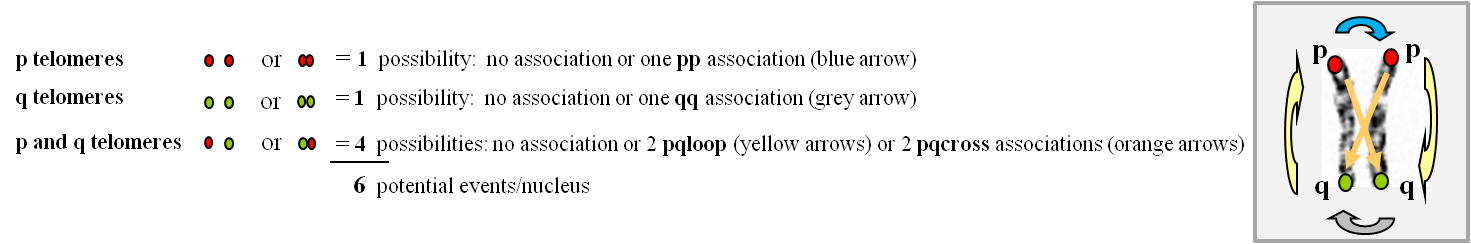
**

**Additional file 8: Figure S4 – Estimation of the number of events (telomeric associations) that can occur in a neutrophil nucleus**
